# Cocaine-Induced Raynaud's Phenomenon: A Case Report

**DOI:** 10.7759/cureus.33604

**Published:** 2023-01-10

**Authors:** Heong Jin C Ahn, Albert Tine III, Arsh N Patel, Samantha A Le, Fulton Defour

**Affiliations:** 1 Department of Research, Alabama College of Osteopathic Medicine, Dothan, USA; 2 Department of Clinical Research and Quality Improvement, Alabama College of Osteopathic Medicine, Dothan, USA; 3 Internal Medicine, Thomas Hospital, Fairhope, USA

**Keywords:** drug induced vasculitis, cocaine use, raynaud disease, raynaud’s phenomenon, secondary raynaud's

## Abstract

We illustrate a notable case of a middle-aged male who presents to a community hospital with left third- and fourth-digit discoloration and pain for the past four days. On presentation to the emergency department, a urine drug screen was ordered which showed synthetic cannabinoids, cocaine, and amphetamines. Initial therapy of nitroglycerin paste, oral oxycodone, intravenous Dilaudid®, and aspirin was started, which resulted in decreased subjective pain. The pathophysiology and mechanism of cocaine-induced Raynaud’s phenomenon (RP) are discussed. Our purpose in putting forward this case is to acknowledge cocaine use as a cause of secondary RP and to emphasize the importance of early recognition to reduce the occurrence of digital necrosis.

## Introduction

Cocaine abuse in the United States among persons greater than 12 years old has been an ever-increasing burden and leads to deleterious health consequences in users [1]. According to the 2016 National Survey on Drug Use and Health, the prevalence of cocaine abuse in 2015 increased by 20% in persons greater than 12 years old [1]. The widespread problem of cocaine abuse and its effects on vascular injury have been described in other studies, but Raynaud’s phenomenon (RP) secondary to cocaine abuse has not been discussed in great detail. 

RP is often a clinical diagnosis in which other differential diagnoses must first be excluded. In addition, the etiology can be characterized as either primary or secondary. A diagnosis of RP requires thorough medical history-taking, evaluation of systemic disease or underlying causes, and diagnostic studies. Examples of laboratory tests include complete blood count (CBC), antinuclear antibodies (ANA) titers, rheumatoid factor, and erythrocyte sedimentation rate/C-reactive protein. Positive or abnormally elevated results may indicate the presence of connective tissue diseases (CTDs). In addition, a nailfold capillaroscopy may be performed to rule out CTDs as a possible etiologic cause. Furthermore, diagnostic imaging tests such as computed tomography (CT) scans may rule out differentials such as thoracic outlet syndrome [2].

In this case report, we identify a man who is diagnosed with secondary RP due to cocaine use. Cocaine is a potent arterial vasoconstrictor that can induce ischemia in peripheral or myocardial tissues. The mechanism of action is described through increased sympathetic response leading to excess norepinephrine at the postsynaptic alpha receptors which ultimately results in arterial vasoconstriction. 

## Case presentation

A 45-year-old male presented to the emergency department with a chief complaint of left-sided third- and fourth-digit pain for the past four days. The pain was described as severe constant dull pain not elicited by any specific movements. He denied any trauma associated with the onset of pain but mentioned three previous months of left extremity second-digit pain. The pain was alleviated with ice and elevation. His past medical history included chronic obstructive pulmonary disease (COPD), coronary artery disease (CAD) with one episode of myocardial infarction, nephrolithiasis, depression, anxiety, suicidal ideations, and essential hypertension. At the time of presentation, he was a current smoker with a 30-pack-per-year history and his UDS was positive for synthetic cannabinoids, cocaine, and amphetamines. His previous occupation was as a coal mine worker.

The patient was admitted from the emergency department for treatment and further evaluation. Vital signs were significant for hypertensive urgency (blood pressure 213/150) and tachycardia. Physical exam was notable for 2+ radial pulses bilaterally, wheezing, anxious mood, agitated behavior, 3/5 strength of bilateral hands, and tenderness to palpation along the third and fourth digits with exacerbation of pain in wrist extension. The patient was stabilized with nifedipine 30 mg po (orally) and metoprolol 12.5 mg IV (intravenous). 

**Figure 1:**
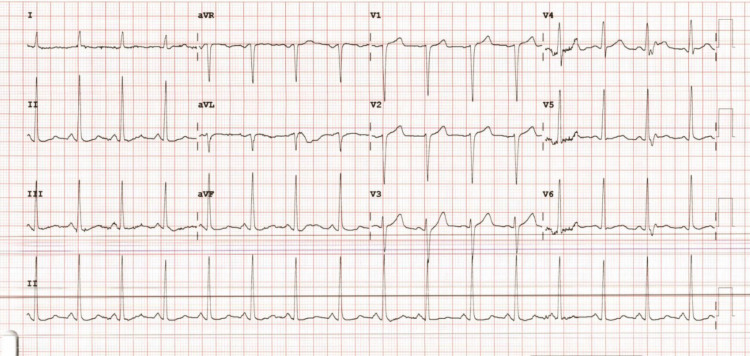
Electrocardiogram for cardiac workup. Normal sinus rhythm with left ventricular hypertrophy.

**Figure 2:**
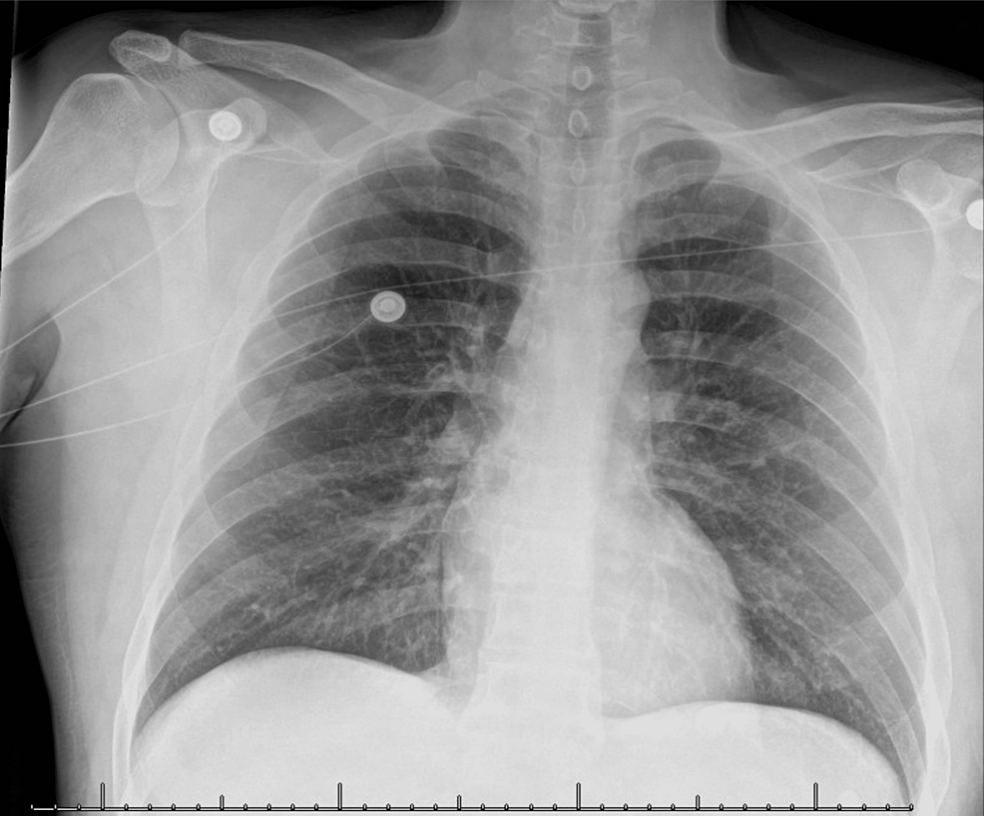
Chest X-ray. Negative chest x-ray showing no acute changes.

**Figure 3:**
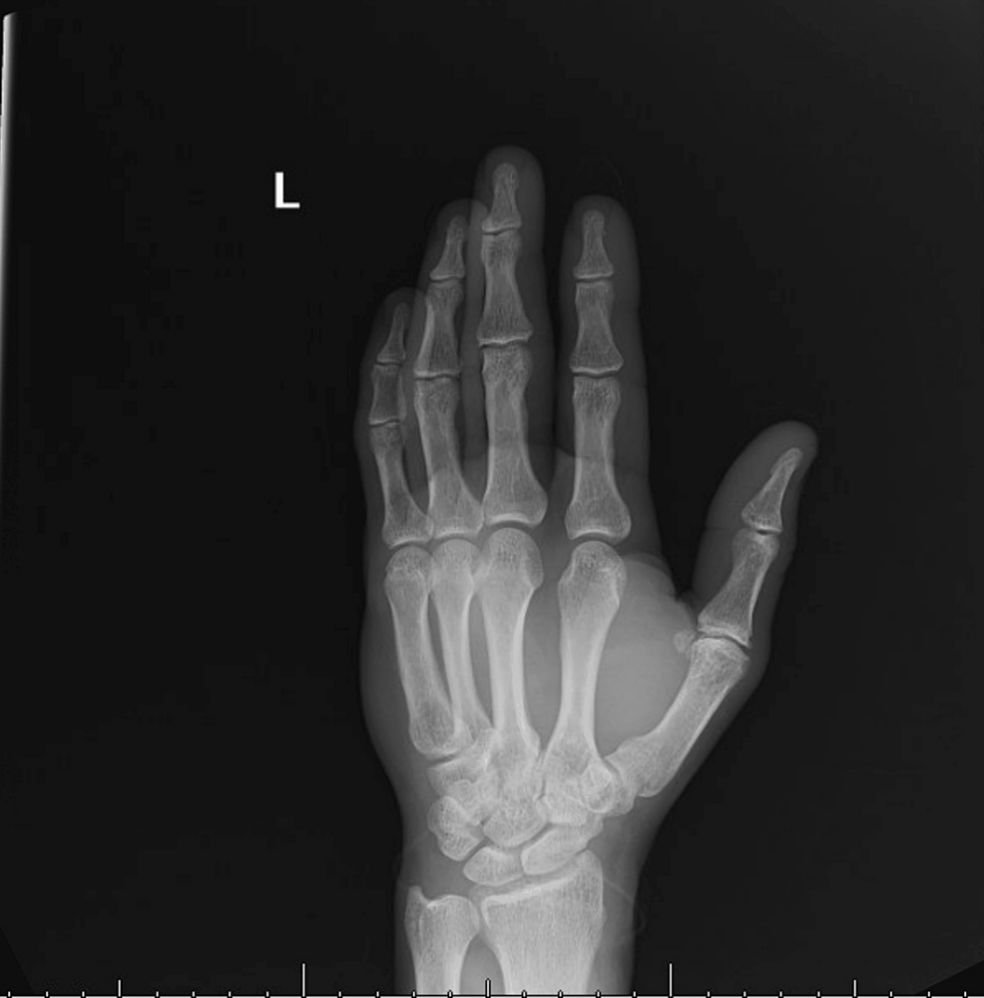
Anterior-Posterior (AP) view of the left hand. Minimal osteoarthritic changes of thumb metacarpophalangeal joint.

An electrocardiogram (EKG) was ordered, which showed normal sinus rhythm with left ventricular hypertrophy (LVH) and possibly Q waves within the anterior leads (Figure 1). Radiographs of the chest (Figure 2) and left hand (Figure 3) were ordered to rule out any acute physical and cardiopulmonary abnormalities. The chest radiograph was negative, and the left-hand radiograph showed minor osteoarthritis of the thumb metacarpal phalangeal joint. A computed tomography angiography (CTA) scan of the left upper extremity and chest as well as a Doppler arterial ultrasound (US) were ordered to evaluate underlying vascular compromise related to ischemic changes observed. The left upper extremity CTA showed complete patency with no acute abnormalities (Video 1), while the chest CTA showed right lobe bronchopneumonia and mild emphysema. No arterial abnormalities were found in the US (ultrasound).

**Video 1:** Upper extremity CTA without contrast Left subclavian artery, axillary artery, brachial artery, radial artery, and ulnar artery are patent. There is no significant arterial stenosis or arterial occlusion seen in the left upper extremity. CTA: computed tomography angiography

Autoimmune panels which included anti-nuclear antibodies (ANA), peri-nuclear antineutrophil cytoplasmic autoantibodies (P-ANCA), antineutrophil cytoplasmic autoantibodies (C-ANCA), and sedimentation rate were negative. Liver studies and cardiac work-up were obtained to evaluate the possible underlying etiology of the digital ischemia (Table 1). 

**Table 1:** Pertinent laboratory data involved in workup.

Lab test	Value	Normal Value
Anti-Nuclear Antibodies (ANA)	Negative	Negative
Myeloperoxidase Antineutrophil Cytoplasmic Antibodies (P-ANCA) Titer	< 1.20 Au/mL	< 19 Au/mL
Proteinase-3 Antineutrophil Cytoplasmic Antibodies (C-ANCA) Titer	< 1.20 Au/mL	< 19 Au/mL
Erythrocyte Sedimentation Rate	2 mm/hr	< 15 mm/hr
Lipase	24 U/L	< 160 U/L
Total Bilirubin	0.3 mg/dL	0.1 - 1.2 mg/dL
Creatine Kinase-MB	3.90 ng/mL	0-5 ng/mL
Creatine Kinase, Total	290 U/L	55 - 170 U/L
C-reactive protein	0.80 mg/L	< 10 mg/L
Troponin	< 0.01 ng/mL	0 - 0.04 ng/mL

Given the patient’s history of cocaine use, his distal digit ischemia was attributed to secondary Raynaud’s phenomenon. The patient was started on nifedipine 30 mg, aspirin 325 mg, and atorvastatin 40 mg. The patient had a previous history of noncompliance with previous medical advice and was lost to follow-up post-discharge. 

## Discussion

Raynaud’s phenomenon (RP) is described as episodic vasospastic attacks of the distal arteries and arterioles. They can be precipitated by low temperatures and emotional stress. The episodes usually last anywhere from 15 to 20 minutes but can be longer and most commonly affect the distal extremities. RP is categorized as either primary or secondary. Primary RP is generally idiopathic with no signs or symptoms, is diagnosed before 30 years of age, and may be attributed to some genetic predisposition. In contrast, secondary RP is diagnosed after 30 years of age and is associated with multiple sources including medications. These medications include beta blockers, caffeine, ergotamine, chemotherapy agents (bleomycin), decongestants, nicotine, stimulants, and cocaine [3]. Other causes include tobacco use, occupational trauma, hyperviscosity syndromes, vasculitides, connective tissue diseases, hypothermia, and peripheral artery disease [2]. 

RP classically presents in a triphasic pattern. The first, or ischemic, phase is characterized by a white discoloration of the skin. This discoloration is induced by initial exposure to the trigger and leads to arterial vasoconstriction and subsequent ischemia and pallor. The second phase is described as the hypoxic phase and is characterized by blue skin discoloration. This color change is caused by deoxygenation of residual blood trapped in blood vessels. Lastly, the third (hyperemic) phase involves reactive hyperemia, which is characterized by red skin discoloration due to restoration of blood flow through the affected area [2].

The mechanism of action behind cocaine-induced secondary RP is primarily caused by the binding of monoamine receptors and transporters. The resulting accumulation of monoamines (dopamine, norepinephrine, serotonin) leads to increased sympathetic activity, which manifests as increased heart rate and mean arterial pressure. Additionally, arterial vasoconstriction is observed due to alpha-1 receptor agonism and typically manifests as ischemic necrosis [4]. Cocaine has also been shown to affect prostaglandin production via increased thromboxane levels, which lead to further vasospasm, platelet activation, and thrombus formation [5]. Various case reports have described acute ischemia in patients with concomitant cocaine use-notably, a 35-year-old woman presented with RP symptoms and after nasal cocaine use [6]. Additionally, a case report presented by Khouri et al. described a 37-year-old man presenting with finger necrosis due to cocaine abuse, which further emphasizes the importance of rapid diagnosis and treatment to prevent limb ischemia [4]. 

Furthermore, concomitant use of nicotine and cocaine may lead to synergistic vasoconstriction and deleterious effects. Moliterno et al. found that patients with cocaine use also endorsed prior tobacco use [7]. Both cocaine and nicotine have been found to affect alpha receptors and promote endothelial dysregulation. By increasing endothelin-1 release and decreasing nitric oxide concentration, cocaine causes loss of endothelial protection on blood vessels and cell damage. In a similar fashion, animal studies have found that cigarette smoking promotes endothelin release and decreased prostaglandin synthesis [8]. 

As primary RP is generally mild, treatment focuses on improving circulation and includes patient education, avoidance of precipitating factors, and warming agents. Conversely, secondary RP is centered toward treating the underlying cause. Several agents are available and include vasodilators, prostaglandin analogues, and calcium channel blockers [9]. 

The most commonly used medications for RP are calcium channel blockers such as nifedipine, amlodipine, and diltiazem. By blocking calcium channels, these agents improve arteriolar vasodilation and flow, leading to an improvement in symptoms. Additionally, nitroglycerin has also been used to treat Raynaud’s symptoms via smooth muscle relaxation and arterial and venous dilation. Nitroglycerin is available in both oral and topical formulations, and was administered to this patient as a paste. 

## Conclusions

We illustrate a 45-year-old male who presented to the emergency department with third- and fourth-digit pain and ischemia. There was no evidence of any autoimmune, infectious, or atherosclerotic processes; however, further evaluation revealed a normal upper extremity CTA but recent use of cocaine. The negative diagnostic tests resulted in the consideration of the diagnosis of secondary RP. The limited published reports in the literature may be due to under-reporting, but this case report aims to add to the current literature. Additionally, we included the traditional treatment of RP and emphasize the importance of abrupt management when the condition is recognized. 
